# Complete Genome Sequences of 11 *Staphylococcus* sp. Strains Isolated from Buffalo Milk and Milkers’ Hands

**DOI:** 10.1128/MRA.01264-19

**Published:** 2019-11-21

**Authors:** Lucas J. L. Pizauro, Camila C. de Almeida, Iman M. Gohari, Janet I. MacInnes, Luiz Francisco Zafalon, Andrew M. Kropinski, Alessandro M. Varani

**Affiliations:** a Department of Technology, Sao Paulo State University, Jaboticabal, Sao Paulo, Brazil; b Department of Microbiology, Sao Paulo State University, Jaboticabal, Sao Paulo, Brazil; c Department of Pathobiology, Ontario Veterinary College, University of Guelph, Guelph, Ontario, Canada; d Brazilian Agricultural Research Corporation (EMBRAPA), Embrapa Southeast Livestock, Sao Carlos, Sao Paulo, Brazil; University of Rochester School of Medicine and Dentistry

## Abstract

Here, we present data on the complete genome sequences of 11 Staphylococcus sp. isolates (three S. chromogenes isolates and one isolate each of S. saprophyticus, S. xylosus, S. hominis, S. agnetis, S. caprae, S. aureus, and S. warneri), obtained as part of a mastitis study of buffalo milk (from healthy animals and from those with subclinical mastitis) and milkers’ hands.

## ANNOUNCEMENT

Like dairy cattle, dairy buffaloes with clinical or subclinical mastitis may have increased somatic cell counts (SCC) and decreased milk production, factors which both have important economic impacts (1). S. aureus is arguably the most important agent of mastitis, although other *Staphylococcus* spp. have also been implicated (2). For example, some coagulase-negative *Staphylococcus* strains are now known to affect the udder of cows and other dairy animals (3). The growing number of genome sequences of *Staphylococcus* species isolated from ruminants with and from those without mastitis is a valuable resource for a better understanding of this important disease (4).

Here, we report the complete genome sequences of 11 *Staphylococcus* sp. strains obtained from buffaloes in Sao Paulo State, Brazil, and from hand swabs of consenting milkers (Table 1). Milk samples were collected after mammary gland physical examination (5), strip cup test, and California mastitis test (CMT) (6) and then submitted for SSC analysis and microbiological culture according to National Mastitis Council guidelines (7). As recommended, a cutoff of 200,000 cells/ml was used to identify subclinical mastitis (8). Milk and hand swab samples were streaked on sheep blood agar and MacConkey agar. Isolates were characterized by matrix-assisted laser desorption ionization–time of flight (MALDI-TOF) analysis at the Animal Health Laboratory, University of Guelph (Guelph, Ontario, Canada), and the identity was confirmed by *cydB* quantitative PCR testing (9, 10). Antibiotic resistance testing (Kirby Bauer) was performed according to the manual of the Clinical and Laboratory Standards Institute (CLSI) (11), and the disk inhibition zones were interpreted according to CLSI guidelines.

**TABLE 1:** Genotypic and phenotypic characteristics of sequenced *Staphylococcus* spp.

Bacterial species	Chromosome or plasmid	Isolate	Size (bp)	No. of genes	Origin	Coagulase positive ornegative^*a*^	CMT^*b*^	SCC (10^3^ cells/ml)^*b*^	Resistance phenotype^*c*^	Resistance gene(s)^*d*^
*S. chromogenes*	Chromosome	34B	2,369,172	2,345	Milk/subclinical mastitis	+	+++	1,254	None	
*S. chromogenes*	Chromosome	17A	2,351,540	2,289	Milk/healthy buffalo	−	−	139	Erythromycin	*tetK*
	Plasmid 1	17A	43,034	59						
*S. chromogenes*	Chromosome	20B	2,424,566	2,417	Milk/subclinical mastitis	−	+	226	None	*blaZ*
*S. aureus*	Chromosome	13	2,737,143	2,830	Milkers’ hands	+			Penicillin and erythromycin	*blaZ*
*S. caprae*	Chromosome	26D	2,662,916	2,645	Milk/healthy buffalo	−	−	16	Penicillin and cotrimoxazole	*blaZ*
	Plasmid 1	26D	28,583	38						
*S. hominis*	Chromosome	19A	2,202,898	2,176	Milk/subclinical mastitis	+	+	251	Penicillin and erythromycin	*mphC*, *msrA*
	Plasmid 1	19A	36,240	46						
	Plasmid 2	19A	42,082	61						
*S. pasteuri*	Chromosome	3C	2,456,297	2,388	Milkers’ hands	−	nt	nt	Penicillin	*blaZ*
	Plasmid 1	3C	110,392	117						
*S. saprophyticus*	Chromosome	1A	2,605,152	2,562	Milkers’ hands	−	nt	nt	Chloramphenicol	*dfrG*
	Plasmid 1	1A	30,637	49						
*S. agnetis*	Chromosome	12B	2,345,021	2,308	Milk/subclinical mastitis	−	+	289	None	
*S. warneri*	Chromosome	16A	2,485,926	2,448	Milk/healthy buffalo	−	−	51	Penicillin	*aaD*, *blaZ*
	Plasmid 1	16A	98,949	139						
	Plasmid 2	16A	76,558	90						
	Plasmid 3	16A	108,569	119						
*S. xylosus*	Chromosome	2	2,797,465	2,697	Milkers’ hands	−	nt	nt	Penicillin	
	Plasmid 1	2	49,406	61						

a +, positive; −, negative.

b SSC and CMT values are from the milk where the isolate was obtained and are not applicable to samples obtained from milkers’ hands. +, weak positive; ++, positive; +++, strong positive; nt, not tested.

c Antibiotics (Kirby Bauer test concentration) tested: cefepime (30 μg), ciprofloxacin (5 μg), chloramphenicol (30 μg), clindamycin (2 mg), erythromycin (15 μg), gentamicin (10 mg), oxacillin (1 μg), penicillin G (10 Un), rifampin (30 μg), trimethoprim-sulfamethoxazole (25 μg), tetracycline (30 μg), and vancomycin (30 μg).

d Antibiotic resistance genes: *blaZ*, beta-lactam resistance; *tetK*, tetracycline resistance; *mphC*, macrolide resistance; *msrA*, macrolide, lincosamide, and streptogramin B resistance; *dfrG*, trimethoprim resistance; *aaD*, aminoglycoside resistance.

Total cellular DNA was extracted using a bacterial DNA extraction protocol (Qiagen, Limburg, Netherlands) with an additional lysostaphin digestion step (12). The quality of the genomic DNA (gDNA) was evaluated by agarose gel electrophoresis and Qubit fluorometric spectrophotometry quantitation. SMRTbell libraries were prepared from gDNA using the PacBio SMRTbell template prep kit 1.0. SMRTbell libraries were size fractionated using a SageELF device (Sage Sciences, Beverly, MA). Genome sequencing was done using PacBio RS II technology at the Génome Québec Innovation Centre (McGill University, Quebec, Canada) with one single-molecule real-time (SMRT) cell per sample. On average, 130,000 reads were generated for each genome (read *N*_50_, 12 kbp), and quality control was performed using FASTQC v0.11.8 software (http://www.bioinformatics.babraham.ac.uk/projects/fastqc). The reads were trimmed and assembled using SMRT Analysis v2.3.0 software, and then assembled contigs were circularized using the minimus2 tool in the AMOS package (13). Trimmed reads were mapped against the assembled and circularized genomes; single-nucleotide polymorphism (SNP) corrections were done with variant-caller software v4.2 in the SMRT package using the quiver algorithm. The genomes were annotated using the NCBI Prokaryotic Genome Annotation Pipeline (PGAP) (14); antibiotic resistance genes were predicted with ResFinder v3.1.0 (15). All software was run using default parameters. To our knowledge, this is the first report of complete genome sequences of *S. chromogenes* and *S. caprae.* The seven strains carrying plasmids have genes for β-lactam (*blaZ*), macrolide (*mphC*), macrolide, lincosamide, and streptogramin B (*msrA*), aminoglycoside (*aadD*), and tetracycline (*tetK*) antimicrobial resistance, which present a possible animal and public health concern.

### Data availability.

Sequence and annotation data of the strains were deposited in the GenBank database under BioProject accession number PRJNA482667 and the BioSample accession numbers SAMN09714551 (*S. chromogenes* 34B), SAMN09714428 (*S. chromogenes* 17A), SAMN09714506 (*S. chromogenes* 20B), SAMN09710868 (*S. saprophyticus* 1A), SAMN09714559 (*S. xylosus* 2), SAMN09714635 (*S. pasteuri* 3C), SAMN09714578 (S. hominis 19A), SAMN09714665 (*S. agnetis* 12B), SAMN09714418 (*S. caprae* 26D), SAMN09714411 (S. aureus 13), and SAMN09714427 (*S. warneri* 16A). Raw sequence data were deposited to the Sequence Read Archive (SRA) and linked to BioProject PRJNA482667.
